# The relationship of *p*50 with clinical outcomes in ventilated preterm infants

**DOI:** 10.3389/fped.2025.1692173

**Published:** 2025-12-19

**Authors:** Ourania Kaltsogianni, Christopher Harris, Stergios Nasikas, Anne Greenough, Theodore Dassios

**Affiliations:** 1 Department of Women and Children’s Health, School of Life Course Sciences, Faculty of Life Science and Medicine, King’s College London, London, United Kingdom; 2 Neonatal Intensive Care Centre, King’s College Hospital NHS Foundation Trust, London, United Kingdom; 3 Neonatal Intensive Care Unit, Department of Pediatrics, University of Patras, Patras, Greece

**Keywords:** blood gas analysis, bronchopulmonary dysplasia, intraventricular haemorrhage, respiratory distress syndrome, retinopathy of prematurity

## Abstract

**Purpose:**

The arterial oxygen tension at which haemoglobin is saturated at 50% (*p*50) can be used as a marker of respiratory disease severity. We aimed to explore whether *p*50 was higher in preterm infants who developed bronchopulmonary dysplasia (BPD) and extrapulmonary complications of prematurity compared to infants who did not.

**Methods:**

Ventilated infants born before 32 weeks of gestation with central arterial access were retrospectively studied. The *p*50 was measured by automated blood gas analysis in the first three days after birth. Outcomes included BPD, intraventricular haemorrhage (IVH), retinopathy of prematurity (ROP) and necrotising enterocolitis (NEC).

**Results:**

One hundred and five infants (50 male) with a median (IQR) gestational age of 26.6 (24.9–28.6) weeks and birth weight of 0.88 (0.68–1.13) kg were studied. They had a median (IQR) *p*50 of 3.34 (3.08–3.77) kPa. IVH was significantly associated with the *p*50 (adjusted *p* = 0.020, Odds Ratio: 2.9, 95% CI: 1.2–7.1) after adjusting for gestational age. The *p*50 was not significantly different in infants who developed BPD, ROP and NEC vs. the infants who did not develop these complications after adjusting for confounders.

**Conclusion:**

Intraventricular haemorrhage in ventilated preterm infants might be associated with an increased *p*50 in the early days after birth.

## Introduction

Acute respiratory disease in prematurely-born infants is typically caused by surfactant deficiency in underdeveloped lungs. The ensuing respiratory distress coupled with invasive ventilation can trigger an inflammatory cascade which contributes to the development of bronchopulmonary dysplasia (BPD) but can also be involved in the pathophysiology of extrapulmonary complications such as intraventricular haemorrhage (IVH), retinopathy of prematurity (ROP) and necrotising enterocolitis (NEC). These disorders share some common pathophysiological mechanisms such as oxidative stress, inflammation and abnormal vascular development following premature birth (1).

**Figure 1:**
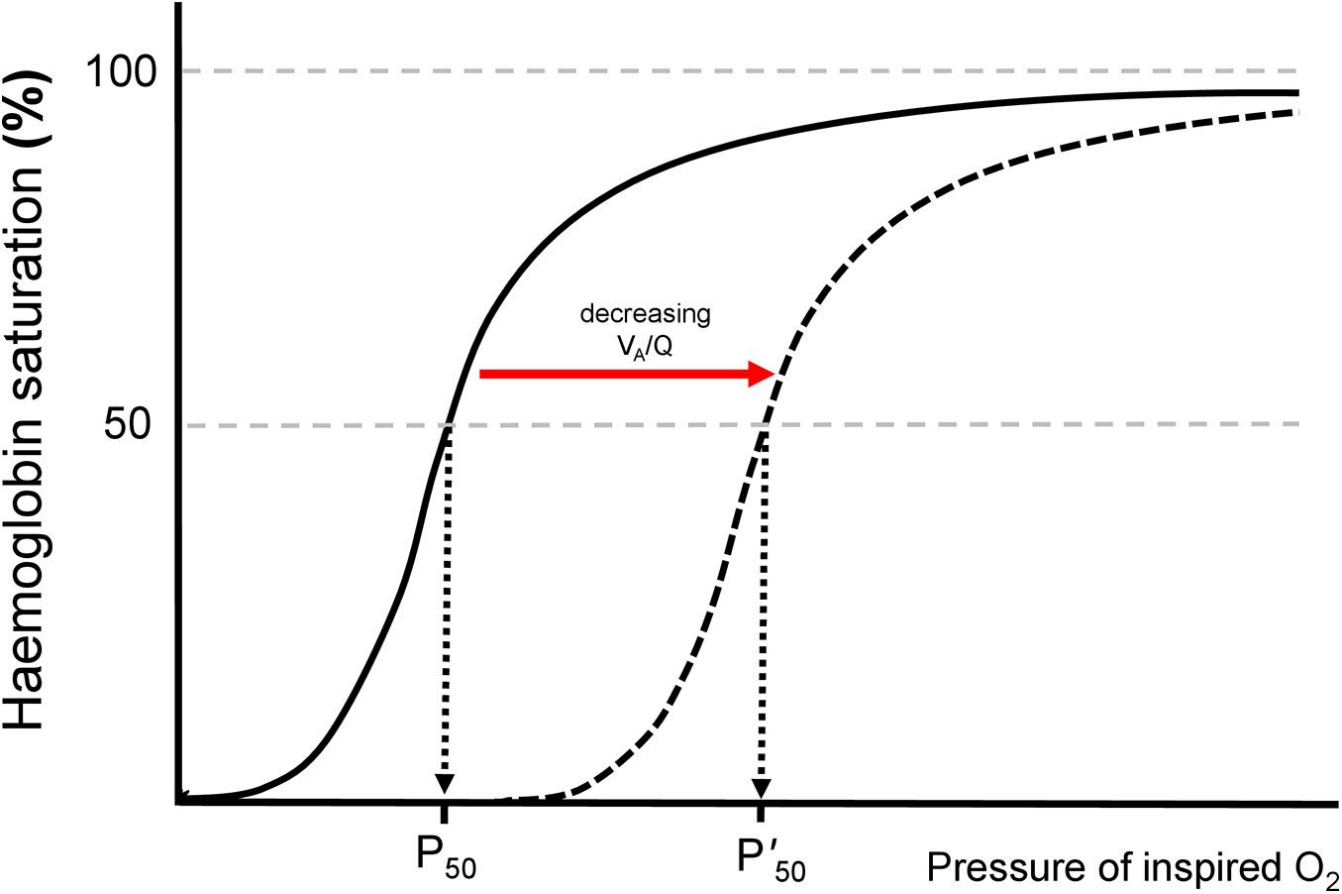
The oxyhaemoglobin dissociation curve. The P_50_ is the oxygen tension at which haemoglobin is 50% saturated with oxygen. When the ventilation to perfusion ratio (V_A_/Q) is decreasing, the curve shifts to the right and the P_50_ increases.

Severe early respiratory disease has been associated with worse extrapulmonary outcomes in preterm infants with respiratory distress. An increase in pro-inflammatory cytokines in ventilated preterm infants has been related to the development of early IVH (2). Furthermore, a longer duration of invasive ventilation, considered a proxy for more severe respiratory disease, has been associated with a higher risk for IVH in preterm infants (3). The presence and duration of mechanical ventilation are valuable clinical parameters, but from the methodological perspective, quantitative indices of respiratory disease severity could better describe the association of respiratory distress with later outcomes. One such index might be the *p*50, the arterial oxygen tension at which haemoglobin is saturated at 50% (4). In concept, right shift of the oxyhaemoglobin dissociated curve secondary to impaired ventilation to perfusion matching, would correspond to a higher *p*50 value (Figure 1). The *p*50 is a component of automated blood gas analysis, which is performed as part of routine neonatal intensive care and is readily available during early postnatal life, as many of the infants with clinically significant respiratory distress will have central umbilical arterial access (5, 6). Despite the above, *p*50 is currently not routinely considered in the clinical assessment of prematurely born infants.

Our hypothesis was that in preterm infants with respiratory distress, *p*50 measured in the early days after birth would be associated with the development of BPD, IVH, ROP and NEC. Our aim was to test that hypothesis.

## Methods

### Study design and subjects

A retrospective, observational study of all infants born before 32 completed weeks of gestation and admitted to the tertiary Neonatal Intensive Care Unit at King's College Hospital NHS Foundation Trust, London, UK between January 2018 and September 2021 was undertaken. Infants were identified via the BadgerNet Neonatal Electronic Patient Record (Clevermed, Edinburgh, UK). Infants with major congenital anomalies, including congenital diaphragmatic hernia or congenital pulmonary airway malformation were excluded. Infants were included if they had arterial access in the first three days after birth and at least five arterial blood gases available for analysis. Infants with pneumothorax who required chest drainage in the first three days of life were also excluded due to the known association of pneumothorax with IVH and BPD (7). The study was registered with the Clinical Governance Department of King's College Hospital. The Health Research Authority Toolkit of the National Health System, UK confirmed that the study would not need regulatory approval by a Research Ethics Committee.

### Information from the medical notes

The following information was collected: sex, gestational age (weeks), birth weight (kg), birth weight z-score (8), full course of antenatal corticosteroids (9), caesarean section (yes/no), duration of invasive ventilation (days), bronchopulmonary dysplasia (BPD) at 36 weeks postmenstrual age (yes/no) (10), patent ductus arteriosus (PDA) medically treated or surgically ligated (11), intraventricular haemorrhage (IVH) grade III or IV (yes/no) (12), age at IVH cranial ultrasound scan (days), blood culture positive sepsis (yes/no), necrotising enterocolitis (NEC) requiring medical or surgical treatment (yes/no), retinopathy of prematurity treated by laser or anti-VEGF (ROP) (13), postmenstrual age at discharge (weeks), weight at discharge z-score.

### Arterial blood gas analysis

Blood gas analysis was performed as part of routine clinical care. Samples were analysed using the ABL90 FLEX PLUS analyser (Radiometer UK Ltd) and results were stored in the archived database on the blood gas analyser hard disk. For each participating infant, the *p*50 from every blood gas was recorded for the first three days after birth and the maximum *p*50 was reported. The standardised *p*50 was calculated as per the manufacturer at a patient temperature of 37 °C, pH of 7.40, partial pressure of carbon dioxide of 5.33 kPa and assuming negligible fractions of carboxy-haemoglobin and meth-haemoglobin. The time period of the first three days after birth was selected as during this period there is consistently arterial blood access in preterm infants with respiratory disease and because more than 90% of the IVHs happen during this time period (14).

### Clinical management

Standard respiratory management of infants born at <32 weeks of gestation was that infants with an oxygen requirement of >40% and/or signs of respiratory distress were intubated, given surfactant and ventilated on volume-targeted, patient-triggered (assist control) ventilation with the SLE6000 neonatal ventilator (SLE, Croydon, UK). The fraction of inspired oxygen was manually adjusted to achieve oxygen saturation levels measured by pulse oximetry (SpO_2_) of 91%–95% (15) and ventilation settings were adjusted to achieve a partial pressure of carbon dioxide of 4.5 kPa to 8.5 kPa for days 1–3 and 4.5 kPa to 10 kPa from day 4 onwards (5). Infants who were ventilated with an oxygen requirement exceeding 40% in the first 6 h of life or required continuous blood pressure monitoring had umbilical artery catheterisation which allowed for arterial blood sampling.

### Statistical analysis

Continuous data were tested for normality with the Kolmogorov–Smirnov test and found to be non-normally distributed and were thus presented as median and interquartile range (IQR). The primary analysis aimed to determine if the differences in the *p*50 were statistically significant in infants who developed BPD, IVH, ROP and NEC compared to the infants who did not, using the Mann–Whitney *U* non-parametric test. The incidences of BPD, IVH, ROP and NEC were examined in infants according to sex, antenatal steroids and caesarean section using the chi squared test. The gestational age, birth weight, birth weight z-score, duration of ventilation, duration of oxygen therapy were compared in infants with or without BPD, IVH, ROP and NEC using the Mann–Whitney *U* test. The independent relationship of *p*50 with each outcome (BPD, IVH, ROP and NEC) was examined using separate binary regression analyses with BPD, IVH, ROP and NEC as outcome variables, and the *p*50 and all other demographic or clinical parameters which were found to be significantly associated (*p* < 0.1) with each outcome as dependent variables. The significance level of *p* < 0.1 in the univariate analysis was used as a preliminary screening threshold to select potential variables for the multivariable model. Multi-collinearity among the independent variables in the regression analysis was assessed by examining a correlation matrix for the independent variables. The relationship of the *p*50 with the duration of ventilation and the duration of oxygen was tested in the infants who survived to discharge using Spearman's rho correlation analysis, to examine whether the *p*50 was related to clinically important continuous respiratory outcomes.

Statistical analysis was performed using SPSS software, version 27.0 (SPSS Inc., Chicago, Illinois, USA).

## Results

During the study period 446 infants under 32 weeks were admitted to the Neonatal Unit at King's College Hospital. Three hundred and forty one were excluded due to absence of central arterial access in the first three days, congenital anomalies or pneumothorax. One hundred and five infants (50 male) with a median (IQR) gestational age of 26.6 (24.9–28.6) weeks and birth weight of 0.88 (0.68–1.13) kg were included for subsequent analysis. Their demographics, baseline characteristics and outcomes are presented in Table 1. The infants had a median (IQR) *p*50 of 3.34 (3.08–3.77) kPa.

**Table 1:** Characteristics of the study population.

Data are presented as median (IQR) or *N* (%).	
Male sex	50 (48)
Gestational age (weeks)	26.6 (24.9–28.6)
Birth weight (kg)	0.88 (0.68–1.13)
Birth weight (z score)	0.28 (−0.39–0.96)
Antenatal steroids	80 (76)
Caesarean Section	49 (47)
Invasively ventilated	102 (97)
Duration of invasive ventilation (days)	24 (4–47)
Duration of oxygen therapy (days)	51 (27–72)
Bronchopulmonary dysplasia	71 (68)
Home oxygen	49 (47)
Patent ductus arteriosus	37 (35)
Intraventricular Haemorrhage grade III or IV	20 (19)
Age at IVH cranial ultrasound (days)	3 (1–5)
Culture positive sepsis	55 (52)
Retinopathy of prematurity	10 (10)
Necrotising enterocolitis	38 (36)
Survival to discharge	94 (90)
Postmenstrual age at discharge (weeks)	40.1 (37.3–45.1)
Weight z-score at discharge	−1.27 (−1.93 to −0.44)

### BPD

The median (IQR) *p*50 was not significantly different in infants with BPD [3.44 (3.14–3.80) kPa] compared to infants without BPD [3.16 (2.90–3.75) kPa, *p* = 0.106].

### IVH

The median (IQR) ***p*50** was significantly higher in infants with IVH [3.83 (3.36–4.13) kPa] compared to infants without IVH [3.28 (3.03–3.62) kPa, *p* = 0.004, Table 2]. The median (IQR) **gestational age** was significantly lower in infants with IVH [25.1 (24.3–26.4) weeks] compared to infants without IVH [27.1 (25.2–28.6) weeks, *p* = 0.009]. The median (IQR) **duration of ventilation** was significantly higher in infants with IVH [40 (22–62) days] compared to infants without IVH [20 (4–44) days, *p* = 0.011, Table 2]. The incidence of IVH was significantly higher in infants without **antenatal steroids** (9 of 25, 36%) compared to infants with antenatal steroids (11 of 80, 14%, *p* = 0.020). IVH was not associated with male sex (*p* = 0.504), caesarean section (*p* = 0.612), birth weight z-score (*p* = 0.874) and duration of oxygen therapy (*p* = 0.190).

**Table 2:** Comparison of the p50 and possible confounders according to a diagnosis of IVH and ROP. Data are presented as median (IQR) or N (%).

	IVH	No IVH	p value	ROP	No ROP	p value
p50 (kPa)	3.83 (3.36–4.13)	3.28 (3.03–3.62)	0.004	3.79 (3.47–4.21)	3.29 (3.07–3.61)	0.018
Gestational age (weeks)	25.1 (24.3–26.4)	27.1 (25.2–28.6)	0.009	24.8 (24.0–25.7)	27.1 (25.0–28.6)	0.004
Birth weight z-score	0.22 (−0.09–1.08)	0.35 (−0.50–0.96)	0.874	−0.08 (−2.20–1.26)	0.35 (−0.30–0.97)	0.298
No antenatal steroids	9 (36%)	11 (14%)	0.020	6 (26%)	4 (6%)	0.014
Duration of ventilation (days)	40 (22–62)	20 (4–44)	0.011	50 (42–69)	24 (5–45)	<0.001
Duration of oxygen therapy (days)	61 (36–88)	47 (25–71)	0.190	110 (68–157)	52 (30–71)	<0.001

Following regression analysis, IVH was significantly associated with the *p*50 (adjusted *p* = 0.020, Odds Ratio: 2.9, 95% Confidence Intervals: 1.2–7.1), antenatal steroids (adjusted *p* = 0.048, odds ratio: 0.321, 95% CI: 0.104–0.991) but not with the gestational age (adjusted *p* = 0.077, Table 3). The duration of ventilation and birth weight were not included in the model due to collinearity with gestational age.

**Table 3:** Multivariable binary regression analyses.

a. Intraventricular Haemorrhage			
	Adjusted p	Odds Ratio	95% Confidence Intervals
p50	0.020	2.89	1.18–7.10
Antenatal steroids	0.048	0.32	0.10–0.99
Gestational age	0.077	0.80	0.62–1.03
b. Retinopathy of Prematurity			
	Adjusted p	Odds Ratio	95% Confidence Intervals
p50	0.174	2.32	0.69–7.83
Antenatal steroids	0.047	0.21	0.04–0.98
Duration of oxygen	0.004	1.02	1.01–1.04

### ROP

The median (IQR) ***p*50** was significantly higher in infants with ROP [3.79 (3.47–4.21) kPa] compared to infants without ROP [3.29 (3.07–3.61) kPa, *p* = 0.018, Table 2]. The median (IQR) **gestational age** was significantly lower in infants with ROP [24.8 (24.0–25.7) weeks] compared to infants without ROP [27.1 (25.0–28.6) weeks, *p* = 0.004]. The median (IQR) **duration of ventilation** was significantly higher in infants with ROP [50 (42–69) days] compared to infants without ROP [24 (5–45) days, *p* < 0.001, Table 2]. The median (IQR) **duration of oxygen therapy** was significantly higher in infants with ROP [110 (68–157) days] compared to infants without ROP [52 (30–71) days, *p* < 0.001]. The incidence of ROP was significantly higher in infants without **antenatal steroids** (6 of 23, 26%) compared to infants with antenatal steroids (4 of 69, 6%, *p* = 0.014). ROP was not associated with male sex (*p* = 0.573), caesarean section (*p* = 0.741), birth weight z-score (*p* = 0.298).

Following regression analysis, ROP was significantly associated with antenatal steroids (adjusted *p* = 0.047, odds ratio: 0.206, 95% CI: 0.043–0.981), and the duration of oxygen therapy (adjusted *p* = 0.004, odds ratio: 1.021, 95% CI: 1.007–1.035) but not with the *p*50 (adjusted *p* = 0.174, Table 3). The gestational age, duration of ventilation and birth weight were not included in the model due to collinearity with the duration of oxygen.

### NEC

The median (IQR) *p*50 was not significantly different in infants with NEC [3.53 (3.08–3.88) kPa] compared to infants without NEC [3.29 (3.09–3.71) kPa, *p* = 0.306].

In the infants who survived to discharge from neonatal care, the *p*50 was significantly related to the duration of ventilation (rho = 0.455, *p* < 0.001) and with the duration of oxygen therapy (rho = 0.221, *p* = 0.033).

## Discussion

We demonstrated that in preterm ventilated infants the highest *p*50 over the first three days after birth was significantly associated with the development of severe intraventricular haemorrhage after adjusting for confounding parameters. We also reported that *p*50 was not associated with the development of any of BPD, ROP or NEC.

Very few previous paediatric studies have explored the relation of the *p*50 with the development of significant complications. Kim and co-workers studied 212 ventilated children with a median age of 4.8–6.2 years diagnosed with acute respiratory distress syndrome and reported that *p*50 increased with an increasing severity of the syndrome and that *p*50 demonstrated a significant association with extrapulmonary organ dysfunction and mortality (16). The authors speculated that a high p50 at the time of diagnosis was associated with mortality via extrapulmonary organ dysfunction (16). This observation is in agreement with our findings of an elevated *p*50, which was also associated with extra-pulmonary dysfunction. To our knowledge no previous study in neonatal medicine has used the *p*50 as an index of quantified respiratory disease severity which could be associated with pulmonary and extrapulmonary complications. In concept, an increased *p*50 has similar pathophysiology with a right shift of the oxyhaemoglobin dissociation curve (ODC) in the context of significant ventilation to perfusion mismatch. A curve which is shifted to the right, because of significant ventilation to perfusion imbalance signalling severe respiratory disease, would naturally produce higher *p*50 values. In this sense, a significant right shift of the ODC has been previously reported in acute and chronic neonatal respiratory disease and is also in agreement with our findings (17, 18).

In our current study, a respiratory index such as the *p*50 was independently associated with the development of a non-respiratory complication such as IVH, while neonatal demographics such as the gestational age were not. We have also previously described that the fluctuation of tidal carbon dioxide during resuscitation of preterm infants, which is also a respiratory index, could predict the development of IVH with an area under the ROC curve of 0.940 (19). These findings imply that IVH in preterm infants is essentially a complication relating to resuscitation and early respiratory care and emphasise the importance of tailored and careful respiratory management in the delivery suite and in the neonatal unit with a view to avoid or minimise such complications. It is also interesting that while *p*50 was significantly associated with IVH, it was not related to any of the other complications we examined. This might be primarily explained by the respective critical time windows for these diseases. For example, it is well known that IVH predominantly occurs in the first three days of life (14) while the pathophysiology of BPD involves a longer process and the earliest meaningful prediction of BPD can only be made at day seven of life (20). In relation to ROP, we acknowledge that the disease is nowadays quite rare and our small single centre population might not had been sufficient to capture this outcome.

The position of the *p*50 as an anchor point of the ODC could be influenced by other parameters such as the pH, the temperature, the levels of carbon dioxide and the relative percentage of foetal and adult haemoglobin at the time of study. The pH, temperature and carbon dioxide however would have limited fluctuation as they are serially monitored to remain within a relatively narrow range according to accepted international guidelines (5). In theory, varying percentages of adult and foetal haemoglobin, and possible blood transfusions might had biased our results as the foetal curve is positioned to the left relatively to the adult subtype, signalling higher affinity with oxygen. These differences could thus produce different values of *p*50 if the measured haemoglobin is predominantly adult or foetal. Including, however, samples only from the first three days of life in our study means that the majority of the circulating blood contains predominantly the foetal subtype and this type of bias would be minimised (21).

We should acknowledge that our population consisted of ventilated infants with central arterial access, as we could not have included preterm infants with minimal ventilatory and oxygen requirements who did not require a central arterial catheter. As such, our population did not include preterm infants with mild or without any respiratory disease. These infants however would have a lower incidence of BPD and IVH, and would thus benefit less by identifying continuous biomarkers which are associated with these complications.

Our study has strengths and some limitations. This was the first study to use the *p*50 as a quantifiable index of respiratory disease to describe the strong association of respiratory instability in preterm ventilated infants with the development of IVH. We used a cohort consisting of more than one hundred ventilated preterm infants and used arterial blood samples which, unlike transcutaneous oxygen saturation, can accurately describe arterial oxygen status across a wide range of values. We should acknowledge as a limitation the single-centre and retrospective nature of our study which might have not been able to capture rare complications such as ROP. Capturing outcomes of such incidence, however, might require multicentre studies which come with their own inherent limitations relating to increased heterogeneity and unpredictable adherence to standardised procedures. It is also worth noting that we cannot infer causality from our study as it is not certain whether severe respiratory distress is causative of IVH, or possibly in some cases it is severe IVH which necessitates invasive ventilation.

In conclusion we demonstrated that a high *p*50 in the first three days of life was significantly associated with the development of intraventricular haemorrhage and that the *p*50 can be used to quantify respiratory disease in ventilated preterm infants with respiratory distress.

## Data Availability

The raw data supporting the conclusions of this article will be made available by the authors, without undue reservation.
